# Pacing stress echocardiography

**DOI:** 10.1186/1476-7120-3-36

**Published:** 2005-12-09

**Authors:** Suzana Gligorova, Marco Agrusta

**Affiliations:** 1 U.T.I.C., Clinica Montevergine, Mercogliano (AV), Italy

## Abstract

**Background:**

High-rate pacing is a valid stress test to be used in conjunction with echocardiography; it is independent of physical exercise and does not require drug administration. There are two main applications of pacing stress in the echo lab: the noninvasive detection of coronary artery disease through induction of a regional transient dysfunction; and the assessment of contractile reserve through peak systolic pressure/ end-systolic volume relationship at increasing heart rates to assess global left ventricular contractility.

**Methods:**

The pathophysiologic rationale of pacing stress for noninvasive detection of coronary artery disease is obvious, with the stress determined by a controlled increase in heart rate, which is a major determinant of myocardial oxygen demand, and thereby tachycardia may exceed a fixed coronary flow reserve in the presence of hemodynamically significant coronary artery disease. The use of pacing stress echo to assess left ventricular contractile reserve is less established, but promising. Positive inotropic interventions are mirrored by smaller end-systolic volumes and higher end-systolic pressures. An increased heart rate progressively increases the force of ventricular contraction (Bowditch treppe or staircase phenomenon). To build the force-frequency relationship, the force is determined at different heart rate steps as the ratio of the systolic pressure (cuff sphygmomanometer)/end-systolic volume index (biplane Simpson rule). The heart rate is determined from ECG.

**Conclusion:**

Two-dimensional echocardiography during pacing is a useful tool in the detection of coronary artery disease. Because of its safety and ease of repeatability noninvasive pacing stress echo can be the first-line stress test in patients with permanent pacemaker.

The force-frequency can be defined as *up- sloping *(normal) when the peak stress pacing systolic pressure/end-systolic volume index is higher than baseline and intermediate stress values, *biphasic *with an initial up- sloping followed by a later down-sloping trend, or *flat or negative *when peak stress pacing systolic pressure/end-systolic volume index is equal or lower than baseline stress values. This approach is certainly highly feasible and allows a conceptually immaculate definition of contractility with prognostic usefulness, but its therapeutic implications remains to be established. Bowditch treppe, assessed with pacing stress, can be used to assess the optimal stimulation frequency and to optimise the patient's chronotropic response in programming rate-adaptive pacemakers.

## Background

High-rate pacing is a valid stress test to be used in conjunction with echocardiography; it is independent of physical exercise and does not require drug administration[1]. Its evolution in the last 20 years started from an invasive (intravenous) right atrial pacing modality, combined with a ionising imaging technique such as radionuclide ventriculography; it moved to a seminvasive modality combined with 2D echo, using transnasal[2] or transoral catheter for transesophageal [3-8] left atrial pacing; and finally evolved to a totally noninvasive modality with external programming in patients with permanent pacemaker for right atrial or ventricular pacing [1,9-11].

There are two main applications of pacing stress in the echo lab: the noninvasive detection of coronary artery disease through induction of a regional transient dysfunction[1,11]; and the assessment of contractile reserve through peak systolic pressure/ end-systolic volume relationship at increasing heart rates to assess global left ventricular contractility[9,12].

### Pathophysiological mechanisms of pacing

Pacing can be atrial or ventricular. The paced chamber is the left atrium in transesophageal pacing and the right atrium or the right ventricle in permanent pacemaker stimulation[1].

**Figure 1:**
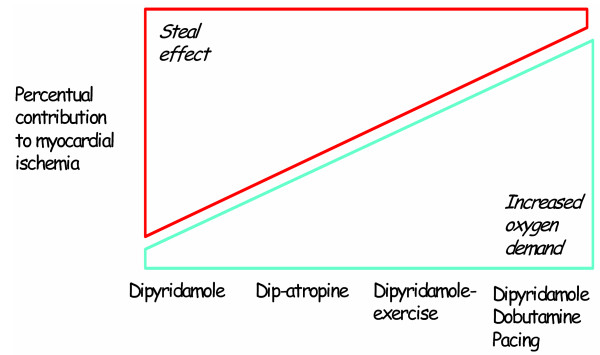
The pathways of ischemia – The pathophysiologic rationale of pacing stress for noninvasive detection of coronary artery disease is obvious with the stress determined by a controlled increase in heart rate, which is a major determinant of myocardial oxygen demand, and thereby tachycardia may exceed a fixed coronary flow reserve in the presence of hemodynamically significant coronary artery disease.

The pathophysiologic rationale of pacing stress for noninvasive detection of coronary artery disease is obvious, with the stress determined by a controlled increase in heart rate, which is a major determinant of myocardial oxygen demand, and thereby tachycardia may exceed a fixed coronary flow reserve in the presence of hemodynamically significant coronary artery disease (fig. 1). With a controlled increase in heart rate during pacing both cardiac volumes and dimensions decreases and blood pressure does not change significantly[1,9].

The drop in subendocardial -to- subepicardial flow ratio[13] associated with rapid atrial pacing in the presence of a tight coronary stenosis, is critical to the development of regional dysfunction, for regional percent systolic thickening is linearly and tightly related to subendocardial, but not to transmural flow[1].

In patients with permanent right ventricular pacing, perfusion defects can often be found in the inferior and apical wall, which are probably the earliest activated sites under right ventricular apical pacing. The regional coronary flow reserve can be impaired in the dominant coronary artery perfusing these regions, whereas it is usually normal in the left anterior descending coronary artery[1]. This abnormality is at least partially responsible for the uncertain specificity of stress myocardial scintigraphy [14].

In patients with permanent pacemakers, chronic right ventricular pacing as a cause of asynchronous electric activation of the left ventricle decreases mechanical load in early versus late activated regions of the ventricular wall. This mechanism induces asymmetric thickness of the left ventricular wall and redistribution of left ventricular mass, with thinning of early versus late activated myocardium[1].

### Septal motion during right ventricular pacing

Variations can be found according to the site of stimulation, the pacing mode and the heart rate[1].

**Figure 2:**
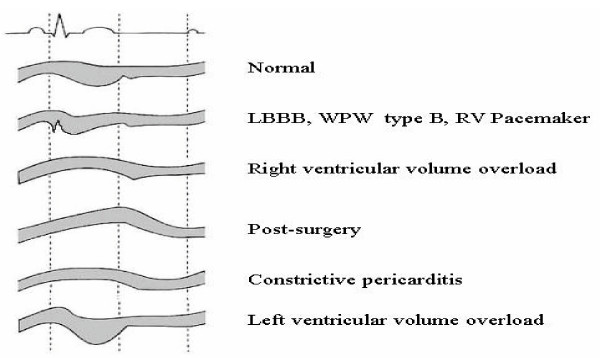
Septal wall motion- extraischemic determinants.

A pre-ejection septal beaking is observed – similarly to what can be found in other patients with relatively delayed left ventricular activation, caused by left bundle branch block or type B Wolff-Parkinson-White syndrome[15] (fig. 2). The pre-ejection period septal beaking is not due to early activation and unopposed contraction of the interventricular septum, but it rather occurs in response to an altered transseptal pressure gradient. When pacing causes the right ventricle to be activated before the left, right ventricular pressure begins to increase in systole before left ventricular pressure, altering the normal left-to-right transseptal pressure gradient. Coincident with the early unopposed increase in right ventricular pressure, the septum abruptly moves posteriorly towards the left ventricle. With the subsequent onset of left ventricular contraction, left ventricular pressure increases, the normal transseptal pressure gradient is restored, and the septum returns in the anterior direction towards its end-diastolic position.

In the ejection phase, a ventricularly paced left ventricle can show a normal posterior motion and thickening (more frequent with pacing from right ventricular apex) or a flat or paradoxical (anterior) motion (more frequent with pacing from right ventricular outflow or right ventricular inflow). The interpretation can be easier in the first case than in the second case, especially considering that in 30% of patients a normal or flat motion can become paradox at high pacing rates over 120/min.

**Figure 3:**
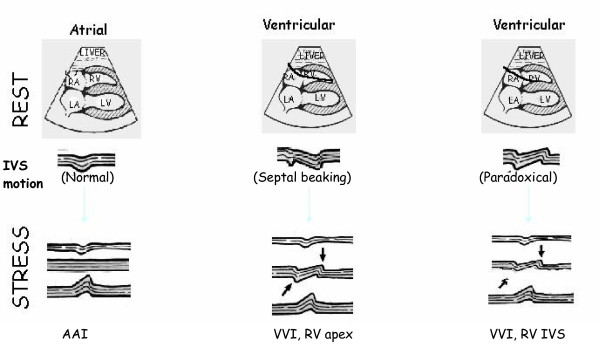
Different types of baseline septal motion and stress-induced ischemia according to the pacing- mode (AAI vs. VVI) and (in VVI) according to the site of stimulation. RV = right ventricle; IVS = interventricular septum.

The interpretation must consider that regional wall motion in the septum is differently affected by the pacing mode(fig. 3). In the atrial stimulation mode, the normal, physiological electrical activation sequence is preserved, and therefore the septal wall motion is normal and there are no special interpretation problems. About two out of three patients with permanent pacemakers are studied in the ventricular pacing mode. In about 30 % of right ventricular-paced patients, the septal wall motion is normal, but in the majority of them an anterior systolic interventricular septal motion (paradoxal motion) is present at baseline. In this case the reader must focus on wall thickening rather than endocardial excursion, and on non-septal regions of the left anterior descending territory to identify left anterior descending stenosis, but this interpretation will always be a challenge, especially at high heart rates [1].

### Techniques of pacing stress echocardiography

#### Intravenous atrial pacing

With intravenous right atrial pacing, diagnostic results are excellent. The technique is, however, invasive since catheterization is required, which nullifies its utilization in the echo lab[1].

#### Transesophageal atrial pacing

The technical possibility of doing transesophageal left atrial pacing[3-8] was suggested more than 30 years ago exclusively for the diagnosis and treatment of arrhythmias. In subsequent years its utilization has been limited by lack of consistent atrial capture and by patient discomfort resulting from high current requirement. Utilization of the transesophageal approach as a stress test for ischemia has become possible thanks to recent improvements in this technique, enabling effective atrial capture at a relatively low threshold, which reduced patient discomfort, and transoral stimulation with 10 French catheters. The transesophageal approach sometimes is ineffective: approximately two patients out of ten have to be excluded either because of pacing-induced chest discomfort not tolerated by the patient, unstable atrial capture, or early appearance of Luciani-Wenckebach second-degree block. To avoid an atrioventricular block during the stress test that will prevent reaching the maximum heart rate, cycle length is progressively decreased to 400 ms prior to performing the continuous pacing of the tests in order to select patients who require atropine sulfate (0.02 mg/kg i.v.) premedication because of a low Wenckebach point[1].

#### Noninvasive atrial pacing in patients with permanent pacemaker

The presence of a permanent pacemaker can be exploited to perform a pacing stress in a totally noninvasive way by programming the pacemaker to increasing frequencies[1,9-11]. This test is especially useful in patients with permanent pacemaker because of the fact that the noninvasive diagnosis of CAD in this patients is an extremely difficult task, since the induced rhythm by right ventricular pacing makes the electrocardiogram uninterpretable and stress scintigraphy is plagued by an exorbitant number of false positive results[1,14].

For the patients with Biventricular pacemakers, in cases when limited maximal programmed rate is present, the test should be done with right ventricular pacing mode, to avoid the submaximal heart rate during the test leading to decreased sensitivity, and increased number of the false negative results respectively.

#### Pacing protocol

**Figure 4:**
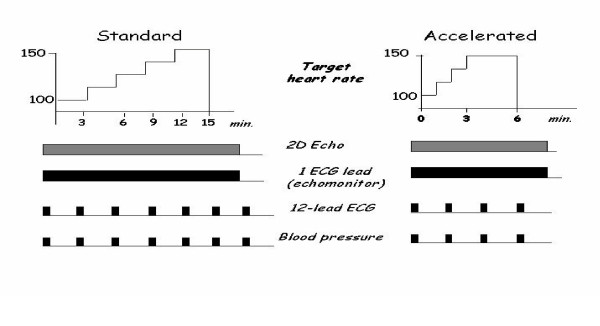
Protocol of pacing stress echocardiography: standard (left) or accelerated (right).

With external programming of the pacemaker, pacing is started at 110 bpm and increased every 2 min by 10 bpm until 85% target heart rate (220-years of age for men; 200-years of age for women) is achieved (fig. 4) or until other standard endpoints (chest pain, Echocardiographic positivity of ischemia, excessive blood pressure fall, limiting dispnea) are reached. The same protocol can also be followed in an accelerated fashion, with faster steps (20 to 30 seconds each) up to the target heart rate. The examination is performed with the patient supine or in left lateral decubitus. Two-dimensional echocardiographic images are obtained before pacing and throughout the stress test, the last recording being obtained after 3 min pacing at the highest rate reached (usually 150 bpm) or target heart rate. Blood pressure and the electrocardiogram are monitored throughout the examination. Left ventricular wall motion abnormalities are evaluated at rest, during pacing, and immediately after pacing interruption[1].

### Diagnostic results

Good diagnostic results have been obtained with invasive atrial, or external (atrial or ventricular) pacing stress echo[11,16]. All stress Echocardiographic diagnosis can be easily summarized into equations, centred on regional wall function, describing the fundamental response patterns: normal, ischemic, viable, and necrotic (table 1).

**Table 1:** Stress Echocardiography in Four Equations

Rest	+	Stress	=	Diagnosis
Normokinesis	+	Normo-Hyperkinesis	=	**Normal**
Normokinesis	+	Hypo,A,Dyskinesis	=	**Ischemia**
Akinesis	+	Hypo,Normokinesis	=	**Viable**
A-,Dyskinesis	+	A-,Dyskinesis	=	**Necrosis**

In the *Normal *response, a segment is normokinetic at rest, and normal-hyperkinetic during stress; in the *Ischemic *response, a segment worsens its function during stress; in the *Necrotic *response, a segment with resting dysfunction remains fixed during stress; in the presence of *Viability*, a segment with resting dysfunction improves during stress. A resting akinesia which becomes dyskinesia during stress reflects a purely passive, mechanical phenomenon of increased intraventricular pressure, and should not be considered as a true active ischemia[10].

**Figure 5:**
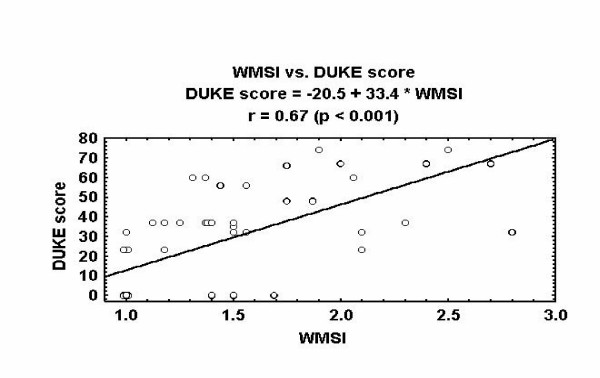
Extend and severity of coronary artery disease (expressed by the prognostically validated Duke score) is predicted by peak Wall Motion Score Index (WMSI) during pacing stress echocardiography.

As with other stress echo tests the positivity can be effectively titrated in the time and space domain: more severe degrees of underlying coronary artery disease are associated with a lower heart rate necessary to induce ischemia and with more extensive wall motion abnormality [17](fig. 5).

A test is considered positive if wall motion abnormalities develop with stress in previously normal territories or worsen in an already abnormal segment[18], (Additional file1 and 2 – Pacing stress negative, Additional file3 and 4 – Pacing stress positive). Another marker of ischemia is reduced regional systolic wall thickening [19].

According to the values derived from the PASE study[11], the use of Noninvasive Pacemaker Stress Echocardiography is diagnostically efficient option for patients with permanent PM and suspected or known CAD (Sensitivity 70%, Specificity 90% and Accuracy 78%).

The *Diagnostic Accuracy *for all stress tests vary widely because there are many factors affecting the test sensitivity. Among the factors that decreases the sensibility are: Absence of previous myocardial infarct[1]; Presence of antiischemic therapy[20]; Stenosis 50–75%, Single-vessel disease, Simple stenosis morphology, Stenosis location LCx, Submaximal stress intensity[1]; Echo interpreter with a low level of competence [18,21,22].

### Limitations

The main limitation of the test is the suboptimal sensitivity in patients with single, and/or mild coronary artery disease, in which wall motion abnormalities may not develop. At a high rate there are fewer video frames during the ejection period and less time to appreciate a regional wall motion abnormality. Only one third of patients can be stressed in atrial stimulation mode that preserves the physiological sequence of contraction of the left ventricle. The external programming of the permanent pacemaker is simple and fast, but it requires technology (external programmer) and expertise, with the need of minimum cooperation and coordination with the pacemaker laboratory[1]. The main source of false negative result is the inability to reach the target heart rate.

### Advantages

Noninvasive pacemaker stress echocardiography has several advantages in comparison to conventional diagnostic techniques[23-25]. The relative merits and limitations of noninvasive pacemaker stress echo versus pharmacological stress echo are reported in Table 2[26-28]. The ability to instantly lower rate and terminate stress result in high test safety[4,29]. Differently from physical stress, it does not require the patient's capability to exercise; differently from pharmacological stress, it does not require an intravenous line and the additional cost (and risk) of drug administration. It also has a shorter imaging time, because the median time of pacing is less than 10 minutes with the accelerated protocol, which compares favourably with the about 15 minutes of infusion time for dipyridamole-atropine and about 25 minutes of dobutamine-atropine. The short duration of pacemaker stress echocardiography and the possibility to perform the test at bedside make it very well tolerated by the patient and user-friendly for the physician[1].

**Table 2:** Pacing versus pharmacological stress echocardiography.

	PACEMAKER	PHARMACOLOGICAL
Modes	Noninvasive PM (transesophageal)	Vasodilation (dob)
Patient tolerability	Very high	High
Stress imaging time	5 to 10'	10 to 20'
Safety	Very high	High
Intravenous line	Usually not required	Required
Echo interpretation	More difficult in ventricular paced	Easier
Clinical experience	Initial	Extensive
Applicability	Pts with permanent pacemaker	All patients

### Pacing stress for contractility assessment through force-frequency relationship

**Figure 6:**
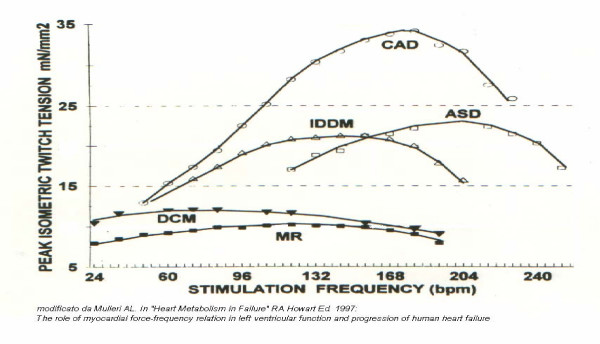
Time sequence during stress. The FFR can be defined as: *upsloping *when the peak stress pacing SP/ESV index is higher than baseline and intermediate stress values; *biphasic *with an initial upsloping followed by a later downsloping trend; *and flat or negative *when peak stress pacing systolic pressure/end-systolic volume index is equal or lower than baseline stress values.

**Figure 7:**
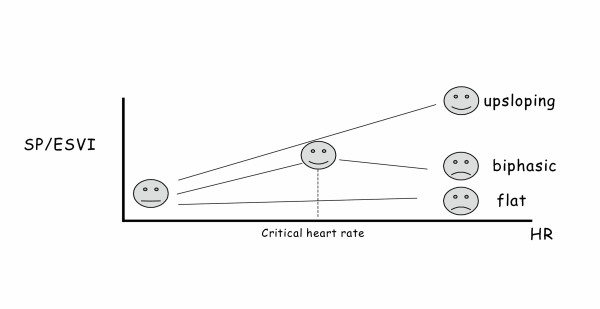
Time sequence during stress. The FFR can be defined as: *upsloping *when the peak stress pacing SP/ESV index is higher than baseline and intermediate stress values; *biphasic *with an initial upsloping followed by a later downsloping trend; *and flat or negative *when peak stress pacing systolic pressure/end-systolic volume index is equal or lower than baseline stress values.

**Figure 8:**
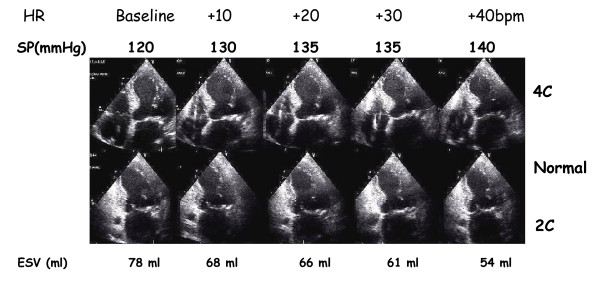
Methodology of the force-frequency curve with pacemaker stress echo in normal subject. On the left, from upper to lower rows: heart rate from external programming of permanent pacemaker (first row); systolic blood pressure by cuff sphygmomanometar (second row); left ventricular end-systolic apical four (4C) and the two (2C) chamber view (third and fourth row); end-systolic volume calculated with biplane Simpson method (fifth row). An increased heart rate is accompanied by an increased systolic pressure with smaller end-systolic volumes (normal up sloping FFR).

**Figure 9:**
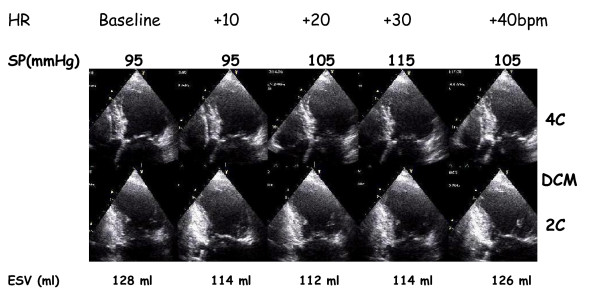
Methodology of the force-frequency curve with pacemaker stress echo in subject with post MI dilated cardiomyopathy and depressed baseline left ventricular function (EF = 30%). On the left, from upper to lower rows: heart rate from external programming of permanent pacemaker (first row); systolic blood pressure by cuff sphygmomanometar (second row); left ventricular end-systolic apical four (4C) and the two (2C) chamber view (third and fourth row); end-systolic volume calculated with biplane Simpson method (fifth row). An increased heart rate at peak pacing stress is accompanied by no change in end-systolic volumes (abnormal flat-biphasic FFR).

Estimating contractility of the left ventricle with non-invasive techniques is an important yet elusive goal[9,12]. The use of pacing stress echo to assess left ventricular contractile reserve is less established, but promising. Positive inotropic interventions are mirrored by smaller end-systolic volumes and higher end-systolic pressures. An increased heart rate progressively increases the force of ventricular contraction (Bowditch treppe or staircase phenomenon)[9,12,30-33]. To build the force-frequency relationship (FFR), the force is determined at different heart rate steps as the ratio of the systolic pressure (SP), cuff sphygmomanometer/end-systolic volume index (ESV), biplane Simpson rule/body surface area). The heart rate is determinate from ECG. The FFR is built off line. The slope of the relationship is calculated as the ratio between Systolic Pressure/End-Systolic Volume (SP/ESV) index increase (from baseline to peak pacing stress)/heart rate increase (from baseline to peak pacing stress). The FFR can be defined as: *upsloping *(normal contractile reserve) when the peak stress pacing SP/ESV index is higher than baseline and intermediate stress values; *biphasic *(patients with positive stress echo or latent left ventricular dysfunction) with an initial upsloping followed by a later downsloping trend; *and flat or negative *(Ischemic or Dilated Cardiomiopathy) when peak stress pacing systolic pressure/end-systolic volume index is equal or lower than baseline stress values[9,12,34] (fig: 6,7,8,9). This approach is certainly highly feasible and allows a conceptually immaculate definition of contractility[9,12,35]; recent studies are reporting its prognostic usefulness[35,36] with a high predictive value of a flat-biphasic FFR for death or acute heart failure; but its therapeutic implications remain to be established.

The critical heart rate (or optimum stimulation frequency) is defined as the heart rate at which systolic pressure/ end-systolic volume index reached the maximum value during progressive increase in heart rate; in biphasic pattern, the critical heart rate is the heart rate beyond which systolic pressure/end systolic volume index declined by 5%; in negative pattern the critical heart rate is the starting heart rate. Abnormal responses are identified on basis of the lower absolute value of FFR slope and of the lower critical heart rate in the presence of abnormal biphasic response of FFR over increasing frequencies. The contraction frequency at which the FFR begins its descending limb ("critical heart rate" or "optimum stimulation frequency") declines progressively with the severity of myocardial disease. Heart rate reduction increases contractile force in end-stage failing human myocardium due to an inverse force-frequency relation. Bowditch treppe, assessed with pacing stress, can be used to assess the optimal stimulation mode (AAI vs DDD vs VVI vs BIV) [37], and the optimal stimulation frequency, and to optimise the patient's chronotropic response in programming rate-adaptive pacemakers[9,12]. More studies are needed to compare the exercise capacity and the clinical outcome in patients programmed to their optimal stimulation rate versus patients who are programmed at more conventional levels.

### Perspectives

As the large and expanding population of patients with permanent PM is present in today's cardiology practice ($2 billion PM in sales, implantation volume ranging from 650 per million in high volume countries, such as Belgium and France, to 200–400 per million in low volume countries such as United Kingdom)[9], the implication of this test is expected to be significantly increased in the future. Beside already proved usefulness in the detection of coronary artery disease[1,11], recent studies suggests extension of the test's utilisation in some other clinical environments and settings: Clinical evaluation of women with suspected CAD[38]; Post transplantation[38]; Assessment of Disease Severity, Risk Stratification, and Prognosis in both Acute Ischemic Syndromes and in Chronic CAD[38]; Before and after revascularisation[38,39], and in Predischarge evaluation [38]; As an alternative diagnostic test for chest pain in elderly[40]; Evaluation of the drug interventions[41]; Pre-operative evaluation in non-cardiologic patients[42]; Patients with new-onset chest pain[43]; In Paediatric patients[44].

## Conclusion

Two-dimensional echocardiography during pacing is a useful tool in the detection of coronary artery disease. Because of its safety and ease of repeatability noninvasive pacing stress echo can be the first-line stress test in patients with permanent pacemakers. The use of pacing stress echo to assess left ventricular contractile reserve is less established, but promising. The force-frequency can be defined as *up-sloping *(normal) when the peak stress pacing systolic pressure/end-systolic volume index is higher than baseline and intermediate stress values, *biphasic *with an initial up- sloping followed by a later down-sloping trend, or *flat or negative *when peak stress pacing systolic pressure/end-systolic volume index is equal or lower than baseline stress values. This approach is certainly highly feasible and allows a conceptually immaculate definition of contractility with prognostic usefulness, but its therapeutic implications remains to be established. Bowditch treppe, assessed with pacing stress, can be used to assess the optimal stimulation frequency and to optimise the patient's chronotropic response in programming rate-adaptive pacemakers. More studies are needed to compare the exercise capacity and the clinical outcome in patients programmed to their optimal stimulation rate versus patients who are programmed at more conventional levels.

## Competing interests

The author(s) declare that they have no competing interests.

## Supplementary Material

Additional File 1 Pacing stress negative, base. Click here for file

Additional File 2 Pacing stress negative, peak. Click here for file

Additional File 3 Pacing stress positive, base. Click here for file

Additional File 4 Pacing stress positive, peak. Click here for file
